# Bittersweet: a multi-method qualitative analysis of Sugar-Sweetened Beverages (SSB) tax discussion in the Nigerian media

**DOI:** 10.1186/s12889-026-28578-5

**Published:** 2026-08-03

**Authors:** Abayomi Tajudeen Sarumi, Opeyemi Ibitoye, Joy Amafah-Isaac, Abdulmumin Opeyemi Muhammed, Abiodun Adebayo

**Affiliations:** 1 Business School (Science Policy Research Unit), University of Sussex, Brighton, UK; 2 Corporate Accountability and Public Participation Africa, Abuja, Nigeria; 3 University of Ilorin, Ilorin, Nigeria; 4 Global Health Advocacy Incubator, Abuja, Nigeria; 5 Economics & Finance Department, Middle Tennessee State University, Murfreesboro, Tennessee USA; 6 Teesside University, Middlesbrough, UK

**Keywords:** Advocacy, Diet-related diseases, Excise, Government representative, Industry actors, Media, NCDs, Public health, SSB tax, Unhealthy diets

## Abstract

In 2021, the government of late President Muhammad Buhari signed the Finance Act of 2021 into law. This law, through the Customs, Excise, Tariffs, etc. (Consolidation) Act, instituted a ₦10-per-litre tax on Sugar-Sweetened Beverages to reduce excessive added-sugar consumption, which has been implicated in non-communicable diseases. This body of work examines the SSB Tax discussion in the Nigerian Media. It identifies critical actors in Nigeria that are impacting the country’s adoption and implementation of an excise tax on Sugar-Sweetened Beverages (SSB). These important actors, government and government representatives, Civil Society Organisations (CSOs) in the country, working on healthy food, public health, nutrition and non-communicable diseases, and players from the SSB industry, which is composed of the producers of these sugary drinks, front groups, and their allies, reflect the understanding gained over time by the general public. The study examined their disposition towards a public health tax levied on a range of products, widely accepted by the public, using Multi-Method Qualitative Text and Discourse Analysis. The research explored media narratives using information from traditional and established media, especially newspapers, for the Nigerian-specific SSB tax narrative, and from a few foreign media outlets for a global perspective. The data gathered was coded, and the results were analysed through Pragmatic-Critical Realism, supported by Subjective Epistemology.

This body of work seeks to fill the widening gap in the media analysis of a critical public health discourse. It provides a foundation for further analysis of the media landscape.

## Introduction

Around the world, there has been growing concern about the rising cases of Non-Communicable Diseases (NCDs). Consuming added sugar is considered a driver of the global increase in overweight and obesity [86]. This unchecked consumption of added sugar and the associated excessive caloric intake stem from sugar-sweetened beverages [44]*.* Generally, unhealthy diets such as Sugar-Sweetened Beverages, excessive salt intake, harmful consumption of alcohol, consumption of tobacco and tobacco products, sedentary lifestyle, etc., are identified as risk factors for different types of non-communicable diseases, including Dental caries, Obesity, type II Diabetes and cancer [54, 69, 70]. The deaths associated with these lifelong diseases in low-and middle-income countries (LIMCs) are increasing, with 72% of these deaths declared as avoidable [61].

In Nigeria, the incidence of non-communicable diseases has been rising, coinciding with dietary changes occasioned by urbanisation and the westernisation of the diet [74]. This rise has deepened Nigeria’s health problem as the country now faces a double burden of disease, with communicable diseases still on the rise and non-communicable diseases killing a significant number of people. In the World Health Organisation’s 2023 edition of the Country Disease Outlook, Malaria, one of the many communicable diseases faced by Nigerians, had an incidence rate of 298.6 cases per 1000 population and resulted in 30% of global deaths associated with Malaria. This rate is similar to the 29% of total deaths associated with non-communicable diseases.

To help countries worldwide combat the scourge of non-communicable diseases, the WHO developed various policy instruments that countries could adopt to reduce the prevalence of NCDs, associated deaths, and identified risk factors [58]. At the top of these tools is taxation on Sugar-Sweetened Beverages. According to the U.S Centers for Disease Control and Prevention (CDC) in 2024, Sugar-Sweetened Beverages (SSB) are any liquids that are sweetened with various forms of added sugars like brown sugar, corn sweetener, corn syrup, dextrose, fructose, glucose, high-fructose corn syrup, honey, lactose, malt-syrup, maltose, molasses, raw sugar, and sucrose. They are generally non-nutritive, contain empty calories [90], and offer no benefits to consumers of any age group [40].

Healthier diets could prevent millions of deaths, reduce healthcare costs for individuals and governments, and put unused revenue back into the economy, thereby enhancing health outcomes. The implementation of policies that expand access to healthy, safe [15], and nutritious foods is also a cost-effective approach to protecting more people from preventable diseases, improving the standard of living and quality of life. The results ripple across all demographics and can be scaled [10, 59, 85].

With clear impacts across all classes of society, the SSB tax is a public health policy that attracts significant public attention, especially from critical players [31], and the media is usually the stage for this attention. This attention is similar to that observed in other public health policies in the same category, including Tobacco Control, Trans-Fat Elimination, and other food-industry-focused policies. A government’s pronouncement of tax on SSB draws a lot of discussion as the government is forced to explain its position and decision. Government officials consistently maintain the tax’s public health rationale, media coverage and news reportage reveal contrasting narratives and themes. Public health campaigners and other Civil Society Organisations like the Corporate Accountability and Public Participation Africa (CAPPA), Centre for the Study of Economics of Africa (CSEA) and other members of CAPPA’s National Sugar Sweetened Beverages Tax Coalition (NSSBTC) demand for stricter tax management including significant increase of the tax rate [92] to comply with WHO recommendations while the producers of SSBs (industry players) [95] and their allies focus on protecting their revenue while warning of immediate and distant economic collapse [28]. Given the considerable impact of media narratives on policy adoption, public understanding, and final implementation, the Nigerian media has not fully explored the effects of narratives and framings of the SSB tax during its implementation and in ongoing advocacy for a review of the tax. This work contributes to the existing body of knowledge by providing insights into the themes and highlighting narratives that will be helpful to public health advocates working within this space.

### Research questions

a How are the identified actors discussing Nigeria’s Sugar-Sweetened Beverages (SSB) Tax in the Media?
b What are the commonly used themes in Nigerian media coverage of the SSB tax?
c In what ways were the issues of public health, economy, and governance/public perception used for or against the policy?

## Literature review

Sugar-Sweetened Beverages (SSBs) are non-alcoholic drinks that contain added sugars, such as regular soda, fruit drinks, sports drinks, energy drinks, and sweetened waters [25]. These beverages are not the same as 100% fruit juice or artificially sweetened drinks. Essentially, any drink with added caloric sweeteners, like high-fructose corn syrup or sucrose, falls under the SSB category [92].

According to UNICEF, these products have adverse effects on children, who are a sizable number of Nigeria’s growing population [75]. While consumption varies by age and geographic distribution, the impact on a global scale is alarming – this global variation, however, has identified patterns including age, sex, and socioeconomic status, with higher intake observed among males, young adults, and individuals in low-income countries [25, 48, 60]. With direct morbidity and mortality linkage, 71% of total deaths in the world are associated with non-communicable diseases and 40% of global disease burden is directly linked to unhealthy diets, including SSBs [45, 94].

A meta-analysis summarising the findings of 32 studies provides overwhelming evidence that an increase in 1 daily serving of SSBs is associated with a 0.22 kg weight gain in adults over 1 year [63]. In addition, there is an increasing body of evidence confirming the causal linkage between the consumption of SSBs and numerous health problems [6], like the increased risk of cardiovascular diseases such as coronary heart diseases and strokes [16], increasing the risk of hypertension [82], and type 2 diabetes and metabolic syndrome [64]. In a 2019 publication, Chazelas et al. noted that in a study of over 100,000 participants who regularly consume SSBs, SSB consumption was linked with increased risk of overall cancer and breast cancer [8, 46]. Data from 2011–2014 show that 63% of young Americans and 49% of older Americans consumed SSBs, with daily average consumption exceeding 140 calories from these beverages alone [25].

Through a systematic review of 17 studies analysing the effectiveness of taxes in reducing SSB consumption and purchases [77], the authors observed that taxation on sugary drinks will affect planned SSB purchases, increasing the likelihood of purchasing available healthier alternatives. It shows that SSB taxes can reduce calories and sugar intake. In the same vein, the work of Teng et al. [87] confirmed that, based on practical assessments of high-income countries classified by the World Bank, taxes on SSBs introduced in these countries were effective in reducing SSB purchases and dietary intake [66].

In 2020, Judit and Guillem estimated that the March 2017 SSB tax regime in Catalonia reduced demand and eventual purchases of SSB by 7.7%, suggesting that a substantial increase in SSB prices due to a properly structured SSB tax will prompt a significant response in sales patterns and volumes [96]. For example, in Mexico, an excise on SSBs was introduced in 2014, with a structure that ensures the tax is passed through to consumers. The study shows greater changes from over-shifting in carbonated SSBs and under-shifting in non-carbonated SSBs [30]. Also, in Berkeley (California), where the SSB tax was introduced in 2015, the 2006 Homescan panel data from Finkelstein et al. [43] estimate that the tax would increase product prices by 20%. Both studies considered the tax impact on SSB consumption and consumption of 12 other food categories. Moreover, they found a reduction of 24.3 kcal per person per day due to the tax, with no substitution by other sugary foods.

The effectiveness of SSB Tax as a policy tool varies significantly, as described by [29], and taxation generally also operates through psychological mechanisms beyond changes in actual costs [71].

Although consumption of SSBs cuts across age groups and demographics, these beverages primarily target vulnerable populations because they are readily available, accessible, and affordable [32, 55, 81]. Similarly, producers of these products devise different means – including aggressive marketing – to exploit information asymmetry and push vulnerable populations into unchecked consumption [24].

In the media, there are emerging roles in shaping policy, particularly in public health. In the lifecycle of public policy formulation and eventual implementation, the media help create awareness, sensitise the public, and, through other modes, including digital media channels, collect feedback to foster a more participatory process [11, 73]. In framing the issues, the media can build support for a successful policy process and, conversely, derail a policy and shut down the policy window of opportunity [38].

SSBs, usually referred to as soft drinks in Nigeria, are a significant source of added sugar and non-nutritive calories in the diet [101], which have been linked to increased risk of type II diabetes, overweight, cardiovascular diseases (CVDs) and obesity [76], and about 15 different types of cancers [26].

For emphasis [5], a Nigerian study of consumption patterns of SSBs in Abeokuta revealed that all undergraduates in Federal Government College of Education (FCE), Federal University of Agriculture, Abeokuta (FUNAAB), and Moshood Abiola Polytechnic (MAPOLY) consumed SSBs, spending between $0.33 – $0.65 on SSB daily. Fadupin et al. [37] also found that soft drink advertising increased consumption among undergraduate students in Southern Nigeria.

After the promotion of Sugar-Sweetened Beverages taxation as an all-inclusive and cost effective [36] policy tool to tackle the rising cases of non-communicable diseases like diabetes and overweight in Nigeria [26], there has been public debates and sectoral discourse observed across different communities of interest on a wide range of topics about SSB tax including but not limited to the effectiveness of the policy, health impacts of consuming SSBs, and unintended consequences of such policy implementation.

Contextually, the efficiency of the SSB tax as a fiscal intervention in public health depends on several factors, including the tax rate. In its initial advisory to member states, WHO suggested that a 20% minimum rate would lead to a pass-through in SSB prices, thereby changing demand patterns. Unfortunately, the tax introduced in Nigeria was only #10 per litre of SSB - a largely insignificant rate. However, in 2025, the WHO commenced its ‘3 by 35’ initiative [100]. This initiative was set up to advance sustainable health financing by increasing taxes on 3 harmful products (Alcohol, Sugary Drinks, and Tobacco) to 50%.

With the government bearing the burden of care in a country with more than 70% of the population paying out-of-pocket [21, 22, 72] for healthcare, the media is also able to play its role as an agent of framing to help the government defend its policy decision, build its own organic support, and lead policy dialogues.

Echoing WHO’s importance of the SSB tax as a policy intervention in a statement credited to the organisation which said ‘SSB taxes can be a win-win-win strategy: a win for public health (and health-care costs that would be saved), a win for the implementing government (increased government’s revenue and improved budgetary allocations for healthcare), and a win for health equity’ (WHO) [99], explained that the taxes on SSBs would benefit from clear definition by the government to avoid interfering lobbyists that can disrupt the set objectives. Experiences from tobacco control advocacy and taxation have shown that lobbying and interference by corporate actors [83], or any antics by business entities, can keep taxes low [84], thereby making it ineffective in achieving public health gains [9].

## Methodology

### Research design

This study employs Multi-Method Qualitative Text and Discourse Analysis (MMTQDA), leveraging the combined benefits of thematic and discourse analysis to explore media representations of the SSB tax in Nigeria by various identified actors. In a 2025 study, De Silva et al. [35] used this method to examine Minority resilience among transgender and gender non-conforming (TGNC) individuals in Sri Lanka. Similarly, this framework was employed by Tian and Dumlao [89] to identify how language learners constructed identities. They specifically used the Critical Classroom Discourse Analysis as an analytical framework with the TA as a method of analysis.

Baker & Levon [12], Liu [62], and Ahn et al. [2] have established that using different approaches in research can benefit the researcher by providing complementary reinforcement. This is a valid justification for the use of mixed methods in helping researchers to make sense of textual data [7], with seemingly no pattern when not analysed. Discourse Analysis (DA) examines the power of language, in both spoken and written forms, to inform and influence the reader [49]. The origin of discourse analysis dates back to ancient Greece, but American philosopher Zane Harris brought it into everyday use in his book Discourse [51]. This is a valid justification for the use of mixed methods in helping researchers to make sense of textual data [7], with seemingly no pattern when not analysed [51]. Also, this approach has been applied across a range of qualitative text and discourse studies and has been advanced as a methodological framework for qualitative text research [7, 67]. Discourse Analysis (DA) is also used to examine the power of language, in both spoken and written forms, to inform and influence readers [49]. The origin of discourse analysis dates back to the ancient Greeks, but American philosopher Zane Harris brought it into common use in his book on Discourse [51]. As part of Multi-Method Qualitative Text and Discourse Analysis (MMTQDA), we combined DA with Thematic Analysis (TA) to codify the text of the various materials studied in this exercise. TA brings deeper meaning to text through identification, assemblage and interpretation [17], mapping themes for more conscious and more rigorous understanding [7].

## Research paradigm

The philosophical foundation of this study provides the basis for understanding the assumptions that underpin the research process and for guiding the interpretation of findings. Accordingly, the research is situated within a Pragmatic-Critical Realist paradigm rooted in Subjective Epistemology. To achieve the desired result for the research questions highlighted below, the ontology for this research work is Pragmatic-Critical Realism rooted in Subjective Epistemology. In the context of analysis and research such as this, pragmatism clarifies the main theories, concepts, and hypotheses under consideration by examining practical considerations to resolve ontological disputes [53].

As part of this methodological framework, we combined Discourse Analysis (DA) with Thematic Analysis (TA), which helped codify the texts of the various materials studied in this exercise. TA adds deeper meaning to text through identification, assemblage, and interpretation [17], mapping themes for a more conscious and rigorous understanding [7]. To achieve the desired result for the research questions highlighted below, the ontology for this research work is Pragmatic-Critical Realism rooted in Subjective Epistemology. In the context of analyses and research such as this, pragmatism clarifies the main theories, concepts, and hypotheses under consideration by examining practical considerations to dissolve ontological disputes [53].

## Data collection

The data for this study were collected through purposive sampling using systematic internet searches. Google and Bing (Microsoft) were used as the primary platforms for searching for Sugar-Sweetened Beverage (SSB) tax media coverage across both traditional and new media. The search was done in the folds.

### Nigeria-focused search

For the Nigeria-focused search, the primary dataset was collated using search terms such as: “SSB tax policy issues in the Nigerian media”, “Sugar-Sweetened Beverages Tax in Nigeria”, “Nigeria’s Finance Act and SSB Tax”, “SSB Tax, media and NCDs in Nigeria”, “SSB Tax Narrative in Nigeria”

### Global search

To situate and contextualise Nigeria’s case within the global discourse, a secondary dataset was developed through internet searches on global discussions using the following terms: “SSB Tax and the media”, “SSB Tax implementation and NCDs”, “SSB Tax policy review”, “SSB tax discourse analysis”

Both Nigeria-focused and global searches were bounded to 2021-2025 to capture narratives and discussions following the enactment of Nigeria’s SSB tax policy. The data were also limited to English-language resources across traditional and digital media, with a focus on policies and academic reviews.

Duplicate articles and other articles that repeated the main messages across the same timelines without contributing any information/insight to the analysis were excluded. While general issues around economic impacts were discussed, unrelated taxation policies or non-health fiscal reforms were excluded.

## Analytical framework

The study employed a dual analytical framework combining Thematic Analysis and Discourse Analysis.

### Thematic analysis

The thematic analysis followed Braun and Clarke’s six-phase approach to qualitative research, which involves: familiarisation with the data, generating initial codes, searching for themes, reviewing themes, defining and naming themes, and producing the report. Adopting this approach provided rigour in identifying recurring themes and patterns in media narratives.

### Discourse analysis

In addition to the thematic coding, discourse analysis was applied to examine how the SSB tax was framed in media narratives. This approach focused on the construction of identities, norms, and perceptions surrounding the policy, drawing on the perspectives outlined by Alejandro and Zhao [7].

## Research question coding

From the search results on Google and Bing, we found a good number of articles and news items that were in line with our search, but also related in content. This is mainly because the same news materials were widely circulated in the media, leading to multiple publications of the same news. Amongst these platforms were Punch Newspapers, The Sun Newspapers, The Daily Trust, and Blueprint Newspapers. Others include Business Day Nigeria, People’s Daily, Nigerian Tribune, The Journal, Guardian, Vanguard, and The Nation Newspapers. The results also referenced similar news reported on multimedia and digital news outlets, including, but not limited to, Aso Radio and TV, News Central Africa, African Independent Television (AIT), and some with only YouTube channels, for news outside Nigeria, Times of India, The Guardian, and Croakey Health Media. It was noted in particular that Health Policy Watch and Croakey Health Watch appear to have a positive slant in their coverage of health-related policies and news. Sifting through articles, 30 articles within the bounded research questions were read for note-taking to identify key messages, language use, image depictions, expert opinions, political views, and interview responses. We also identified claims and possible biases for our analytical coding, which was analysed for themes and patterns.

We fed the NVivo analytical tool with the identified keywords, which were organised into thematic and discourse nodes. See Table 1 below for keywords deployed in the nodes.

**Table 1:** Nodes and Keywords used in NVivo

Thematic Nodes		
	**Node**	**Description/keywords**
1	Public Health Advocacy	Non-communicable diseases, health crisis, out-of-pocket expenditure, diet-related diseases, SSB consumption burden,
2	Profit and Economic Promotion	Price hike, job loss, inflation, sugar sector failure,
3	Policy engagement, corruption, and governance	Policy disconnect, tax earmarking, fiscal policy, revenue generation, health budget, and the effectiveness of the existing rate. What research is available for Nigeria, and what is the best rate?
4	Stakeholder mobilisation, engagement and framing	What are NGOs/CSOs and advocates saying? What is the position of beverage makers and their allies? Is the government speaking, and what is the media saying?
5	Media	Journalism training, media interviews, coalition media engagement, and social media mentions. Does media play a role in policy?
**Discourse Nodes**		
1	Social good	SSB tax is pro-health, pro-people. Protecting vulnerable groups and increasing funds for health to shield the poor
2	Moral Framing	SSBs are portrayed as harmful and disease-causing/life-threatening, but the industry says SSBs are good for life for consumers
3	Urgency and Crisis Language	Urgency in action, health collapse, future burden, and disease transitioning. Comparison with tobacco danger
4	Authority and global best practices	Referencing the World Health Organisation, the Bloomberg Health Task Force and other global voices supporting the SSB tax
5	Economic blackmail	Industry resistance, economic downturn and any information by manufacturers/industry players and their allies speaking in opposition to the SSB tax
6	Business collapse	Claiming that the SSB tax will impact different sectors, including farmers and other sugar-related businesses

## Results

The results of the NVivo analysis are presented in two main sections: Thematic Analysis and Discourse Analysis. These sections detail the primary subjects of discussion and the argumentative styles employed by the key stakeholders, Advocates and Public Health (APH), Industry and Allies (IA), Government and Policy (GP), and Media Source Reporting (MSR). In Table 2 below, the themes are organised through the thematic network.

**Table 2:** Thematic Network for Findings

Global Theme: **Analysis of Sugar-Sweetened Beverages (SSB) Tax Discussion in the Nigerian Media**	
**Organising Themes**	**Basic Themes**
Advocates and Public Health (APH)	The issue is framed as a moral imperative to save lives from NCDs and uses strong, emotive language with a sense of urgency Authority Framing: To support their claims by linking recommended tax hikes (e.g., 50%) to WHO guidelines
Industry and Allies (IA)	They focused on rebutting the Advocate's claims and protecting the industry’s image, including its revenue and doomsday messaging. (See Discourse findings for more details) Authority Framing: To refute health claims by citing other national or international statistics, such as per capita consumption being below WHO recommendations
Government and Policy (GP)	References are mainly neutral statements about signing or implementing policy. Acknowledging health challenges without blaming IA
Media Source Reporting (MSR)	Neutral reporting of WHO recommendations and international precedent (Mexico, Chile) Authority Framing: Authentication of stories and objectivity

*Source: NVivo Data Analysis of Media Links*

### Thematic findings: key areas of contention

The findings from the aggregated thirty (30) news links show five (5) dominant themes in the media narratives used in the argument for the tax and its opposition. Public Health Advocacy and Profit and Economic promotion are more prominently used than stakeholder mobilisation, engagement, and framing. The themes of policy engagement, corruption, governance, and media were least deployed. Interestingly, though the least, they were deployed alongside the most prominent themes. Advocates and Public Health (APH), Industry and Allies (IA), and Government and Policy (GP) were the most prominent stakeholder groups and pillars of the much-conflicted public conversation.

#### Public health advocacy and its advocates

Public Health Advocacy, which primarily concerns interventions with population-wide impact, is conducted in different ways, including community engagement and mass media messaging. Public health further intensifies the science and practice of promoting and protecting population health.

As seen in different media reports, commentaries, and articles, public health advocacy and public health leaning issues were used mainly by Civil Society Organisations (CSOs) like Corporate Accountability and Public Participation Africa (CAPPA), Centre for Study of Economies of Africa (CSEA), and other organisations, including members of the National Sugar Sweetened Tax Coalition (NSSBTC) led by CAPPA. They drew mainly on the burden of disease in Nigeria and the exacerbating problem of Non-Communicable Diseases worldwide, bringing local and global data into the mix. Their framing heightened the sense of danger, showing the progression of public diseases and changes in disease patterns. For example, they shared WHO’s NCD data, *“In 2018, Non-Communicable Diseases (NCDs) were estimated to account for 24% – 29% of all deaths in Nigeria. NCDs account for 24% of total deaths, and cardiovascular diseases take the lead of 11% (over 7%) of deaths attributable to NCDs* [98]*.*

Examining those who rallied support for the government in its implementation of the SSB tax policy, there is a noticeable, strong framing and narrative built around public health, earmarking for the nutrition programme, building a stronger healthcare system, industry interference, and the deliberate targeting of the low-income demographic. Their argument also included a demand for an increase in the tax and for its expanded coverage.

Through news reports, press statements, and articles, they reinforced the general message of health problems, using it as a foundation for their arguments. Advocates utilised strong framing to connect SSBs directly to mortality and health crises, as seen in the statement: *"These sugar-sweetened beverages… are killing us slowly, turning our streets into graveyards and our hospitals into crowded waiting rooms.”*

*“The high rates of obesity, diabetes, hypertension, and other cardiovascular diseases highlight a health crisis in Nigeria, with over 11 million Nigerians currently living with diabetes, he (Akinbode Oluwafemi) disclosed* at a journalism training organised by his organisation to equip journalists with the right information on the policy intervention. ‘Name-calling’, chronic diseases that send shockwaves into public minds, were also deployed in the public health themes. In this area: *“Cancers (3%), diabetes (2%), and chronic respiratory diseases (1%). The probability of dying prematurely from NCDs in Nigeria is over 20% according to the World Health Organisation (WHO)”* was used.

The news coverage of the government’s plan, approval, and implementation of excise taxes on sugary drinks was a sign that the Federal government of Nigeria was concerned about the rising cases of NCDs in the country and believed that the ten-naira-per-litre tax on sugar-sweetened, carbonated, and non-alcoholic drinks could help reduce the menace [27]. While this tax rate was largely insignificant – a consensus amongst public health advocates and health policy experts—it signalled that the government was willing, ready, determined, and committed to tackling the upsurging health crisis through the adoption and implementation of the tax. Government representatives were also repeatedly quoted as referencing the health problems linked with consumption of SSBs as the primary motivation for the introduction of the SSB tax. They further described it as an important policy thrust for the 2021 Finance Act [33]. The government’s use of this framing further showed that the tax’s primary argument was framed in public health terms.

This (SSB Tax) is to ‘discourage excessive consumption of sugar in beverages, which contributes to several health conditions, including type II diabetes, overweight, and obesity.’ [1].

Other quotes of government officials and representatives include, but are not limited to, (a) ‘the tax became inevitable to discourage excessive consumption of sugar in beverages, which had continued to imperil the lives of Nigerians.’, (b)’the implementation of the SSB tax was a strategic move to reduce various health risks.’ ‘We are committed to attaining the global best practice of at least 20% of the final retail price on all SSBS, as the current 10 naira per litre price fails to achieve that.’ [39, 41, 42].

When industry players deploy public health arguments, they primarily use them to deny the problem, shift blame for disease causation to other factors, and claim objectivity and balance. In 2023, the Manufacturers Association of Nigeria (MAN), through its chairman, said SSB is not a problem for non-communicable diseases. ‘According to health experts, a balanced nutritional approach, which allows for the occasional indulgence in sugary beverages, can harmonise with a healthy lifestyle and dispel misconceptions about their influence on obesity and related health issues,’ he noted. The association continued to say, *“experts also stated that the surge in obesity cannot be solely attributed to sugar; inactive lifestyles, lack of physical activity, and overall poor dietary choices play a significant role in the obesity epidemic” as a way to defend its position further.*

**Fig. 1:**
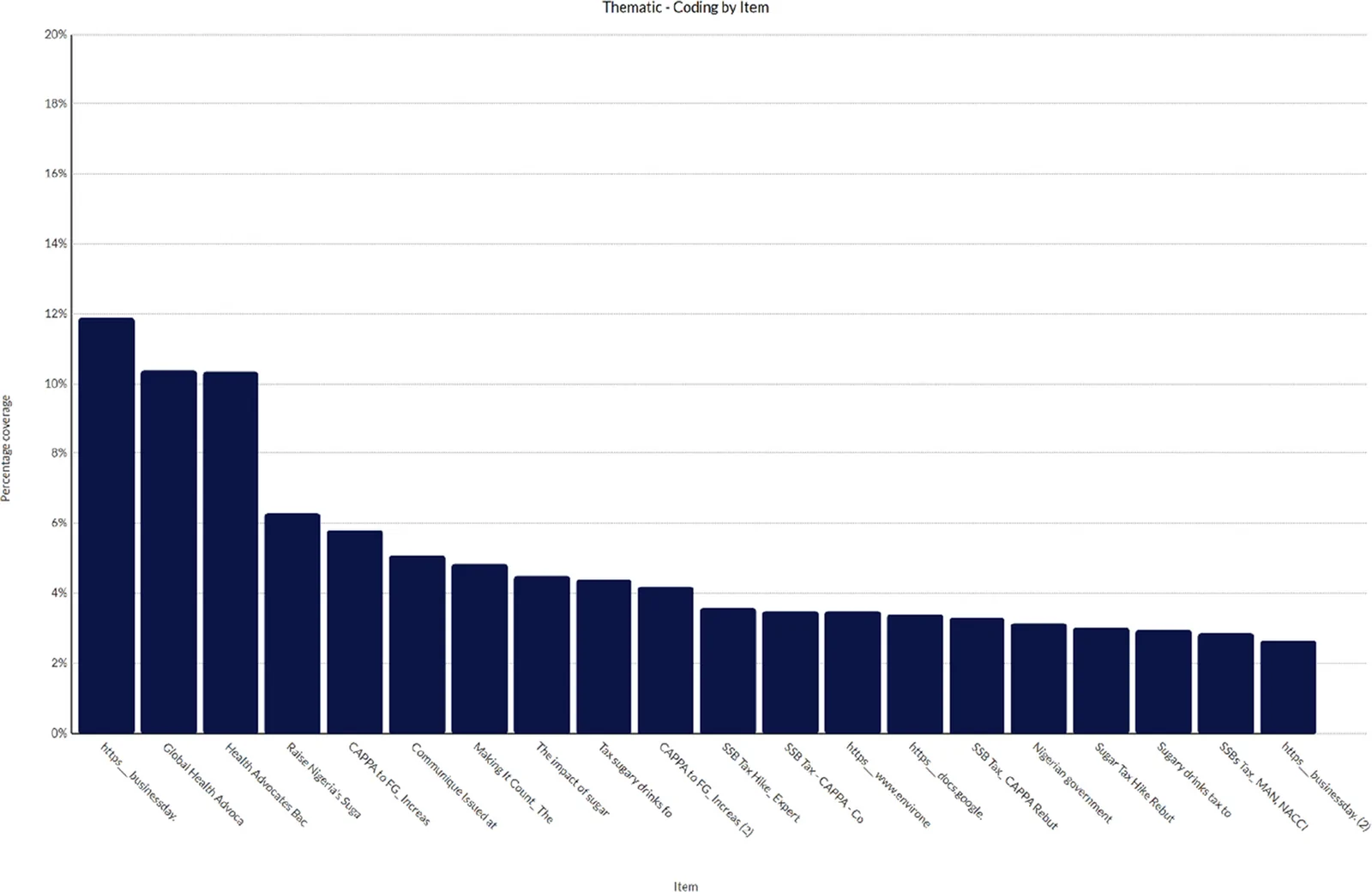
Thematic Coding Coverage by Item. *Source: NVivo Data Analysis of Media Links*

Establishing the possibility of intentional distortion of the public health problem, advocates addressed it by reinforcing the disease burden. *‘We are taken aback by the industry deliberately avoiding the statistics of Nigerians who have diabetes, obesity and other illnesses associated with the products they produce’,* [23]. Figure 1 below shows the average coverage of coded themes.

#### Profit and economic promotion

Under this framing, the industry (sugar-sweetened beverage manufacturers) and its allies focused more on profit and economic growth. They vehemently argued that profit and economic growth were critical, claiming that the industry has been a major contributor to Nigeria’s economic growth, pays huge taxes, and could collapse due to the SSB Tax. The industry’s argument and its allies’ largely revolved around confrontation and defensiveness. MAN Firebacks at NGOs Advocating for SSB Tax Hikes".

Quoting industry-wide data, they claimed that ‘the economic turbulence experienced in 2022 forced the manufacturing sector to record negative growth of −1.91 per cent in *Q3 of the year. The contribution of the manufacturing sector to the GDP also declined from 8.96 per cent in Q3 2021 to 8.59 per cent of real GDP in the third quarter of 2022.’*

Without recourse to numbers directly linked to the SSB manufacturers, the industry and its allies further expanded on the theme of profit and economic promotion by citing public forecasts of possible unemployment in Nigeria. Global audit and tax advisory firm KPMG has projected that Nigeria’s unemployment rate will rise to 40.6% from 37.7% in 2022.

‘As expected, such an outcome would only translate to the loss of millions of Nigerian jobs that are generated by the sector each year.’

Further, they argued that ‘in 2021, when the sugar tax was first implemented, the manufacturing industry suffered major setbacks, with as much as an 8% to 10% decline in profit, with the food and beverage sub-sector experiencing negative GDP growth.’

On the flip side, when public health advocates use themes similar to profit and economic promotion, they present them as national rather than sectoral benefits – usually citing national productivity, lower healthcare costs for individuals and the government, and overall financial benefits. This is a stark contrast to the industry’s defensive argument.

#### Others: complementary themes

The Stakeholder mobilisation and engagement, Policy (debates and analysis) engagement, corruption, governance, and the Media, as identified themes, showed that government, public health advocates, and the industry all deployed these themes alongside their main arguments for public health and economic promotion. From policy actors to government officials, everyone rallied the media to shed light on the topics of concern, feeding them narratives, including data, that suit each’s argument. In its neutrality, media coverage focused heavily on the policy’s technical and mechanical aspects. Key discussions focused on the mechanics of tax rates and price elasticity.

Public health advocates commended the government’s action. However, they demanded a review of the tax structure, including its definition, coverage, and tax rate, and further called for government engagement with additional stakeholders. They reinforced this with arguments based on global best practices on such tax rates, as seen in many media sources reporting on supporters of the tax: *“The overall reduction in quantity demanded of SSB could be negligible if the tax rate does not increase the price by at least 20%.”*

This meant that the themes of governance and corruption were also used to strengthen the argument for the tax, its retention and increase. Salau [80] quoted Abayomi Sarumi in a Guardian newspaper publication, *“We want the government to put the revenue generated from the SSB tax to the treatment of other public health conditions. I am appealing that you all support the government’s initiative on the tax policy by seeing it as a pro-health and pro-life initiative.”*

**Fig. 2:**
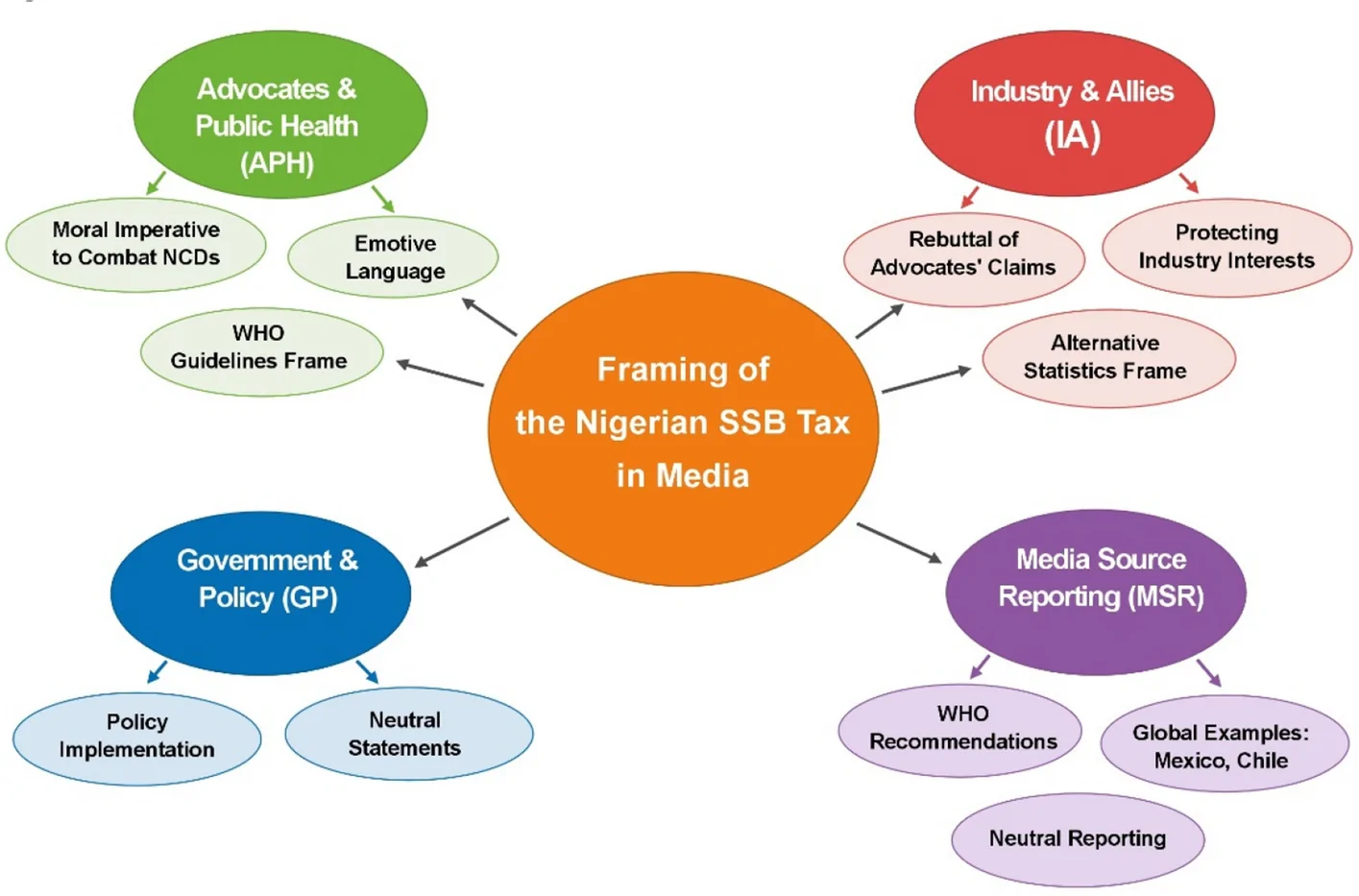
Thematic Network. *Source: NVivo Data Analysis of Media Links*

Arguments like the ‘need for a multisectoral approach’, ‘tiered sugar taxes’, and ‘tax rates and price elasticity’ were usually seen from the industry’s perspective. They further stated that ‘the beverage industry is calling for a broader, more holistic, multisectoral approach to combating the challenges of obesity and other non-communicable diseases. The linkage of themes is presented in Fig. 2.

### Discursive elements: divergent imagery and argumentative frames

The discourse analysis clearly separates the communication strategies of the three main participating stakeholder groups. The most frequent discourse codes were Authority and Global Best Practices, Moral Framing, and Social Good.

#### Authority and global best practices: an equally contested space

In using the authority frame and global best practices, the media further maintained its neutrality by reflecting the discursive elements in arguments for and against the tax. The media referenced WHO recommendations and international precedent (Mexico, Chile). Similarly, public health advocates cite authorities, mainly the WHO and, sparingly, UNICEF and Bloomberg Philanthropies, to defend global best practices regarding an efficient tax rate and other accompanying government policies that will enhance the proposed health gains of the policy intervention.

As industry allies and representatives dismissed public health authorities in their arguments, they repeatedly cited the WHO’s per capita sugar consumption data. For example, MAN noted that *"Nigeria’s per capita consumption of sugar is 7.1 kg… well below the WHO recommended 9.1 kg and the global average of 21.4 kg,"* and therefore asserted that the tax is unnecessary.

#### Moral and crisis framing: the advocate’s trusted weapon

The analysis showed that moral framing was used alongside Urgency and Crisis Language to provide a clearer picture of the situation. This explains why the stakeholder group that uses this discursive element most is public health actors and advocates. They employed strong, visceral language to create a sense of moral duty, linking SSBs to direct harm: *"These sugar-sweetened beverages… are killing us slowly…".* Using phrases like *"public health crisis"* and *"ticking time bomb"* to demand immediate government intervention.

**Fig. 3:**
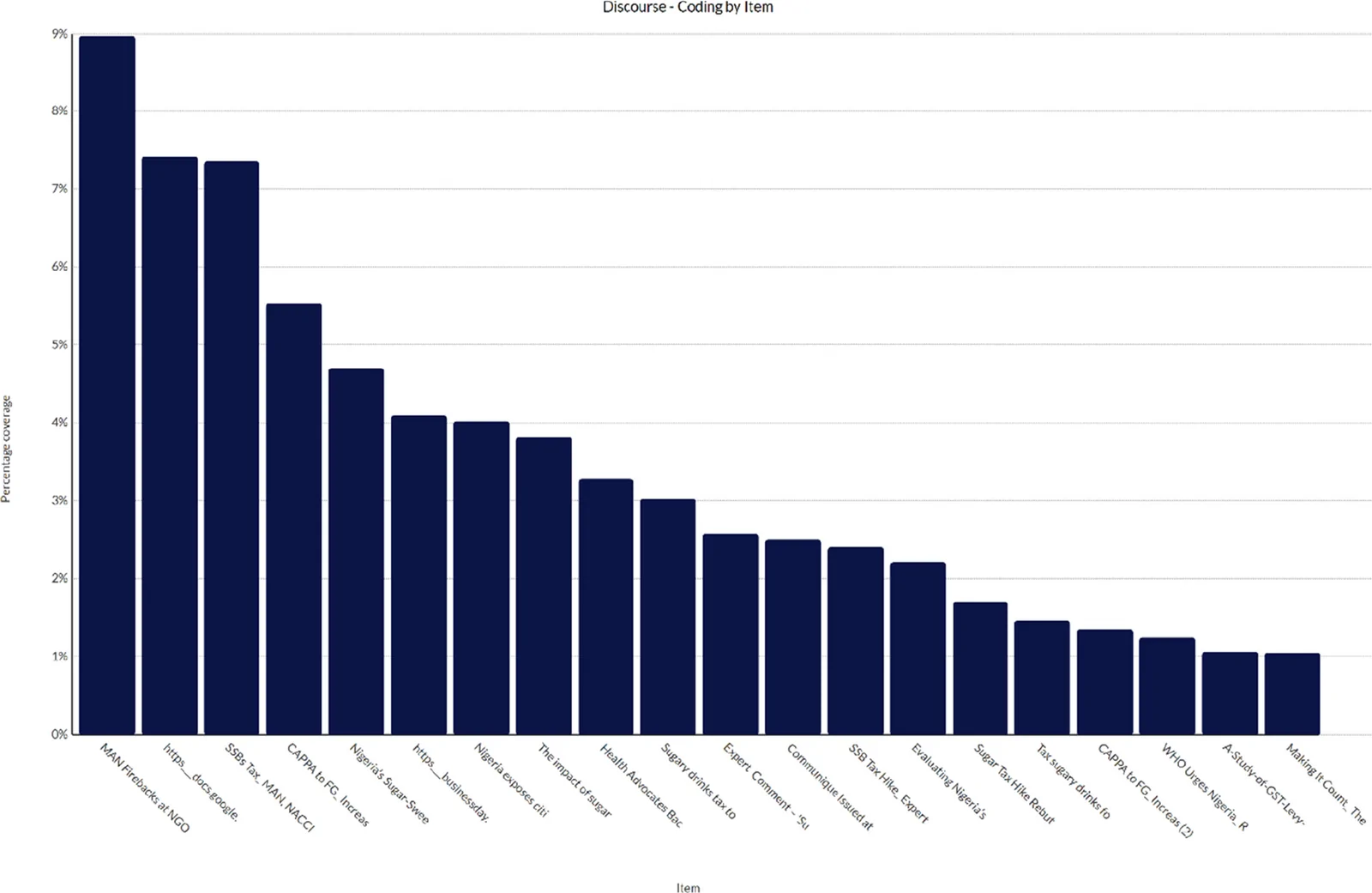
Discourse – Coding Coverage by Item. *Source: NVivo Data Analysis of Media LinksR*

However, when Industry allies deploy this element, they tend to combine it with economic blackmail and threats of business collapse – not just against SSB producers but as an industry-wide problem for Nigeria’s manufacturing sector [88]. In such instances, they dismiss the urgency raised by public health advocates, calling it "alarmist narratives that are often grounded in misleading data- designed to instil fear." The industry representatives further suggested that implementing the new tax could result in nearly 15,000 job losses (direct and indirect) among casual and contract workers [68]. They dramatised the country’s already high unemployment rate at 33%. The percentage distribution of the discursive element is shown below in Fig. 3.

#### The discourse of social good

The Social Good frame was the third-most-frequently deployed discursive element, primarily adopted by the Media and Advocates. The use of social good helped the advocates supporting the tax reflect the general needs of the people, advancing social protection for all citizens, regardless of their status, with greater emphasis on vulnerable groups. Through this, they intensified the ‘national health outcomes’ argument, as captured in statements such as *"…a more robust SSB tax would reduce the consumption of sugar-laden drinks, lower the incidence of preventable illnesses, and improve national health outcomes."*

As they leaned into this element, a subtle use of legacy framing was directed at the country’s government, with the president as the primary target. Public health advocates noted that Nigeria has an opportunity to step ahead of other African countries by setting a robust and effective SSB tax framework capable of delivering public health gains, using the president’s commitment in his manifesto as ‘bait’. As often seen in the pattern of findings, the industry, on the other hand, uses an economic-social-good argument focused on socio-economic well-being: it counters the tax as risking the "likelihood of thousands of Nigerians who depend on the sector."

## Discussion

The study of the media framing of Nigeria’s tax on sugar-sweetened beverages by the identified primary actors, being the government and government agents, Civil Society Organisations and advocates working on public health, and the sugary drink industry and its allies, between the years 2021 and 2025, found that the arguments and discussion on the tax were in two parallel folds. There is an argument building support for or arguing against the tax. There was also a broad representation of government, CSOs, and manufacturers of sugary drinks in the media, with the government and its agents using identical narratives. At the same time, CSO representatives are largely public health advocates.

While this research is qualitative, focusing on language use and the presentation of issues, a pattern emerged in media releases. This correlates with the findings of Buckton et al. [19], who found that more media materials supporting and opposing narratives were deployed around the budgeting period, which could be considered a policy window, and during festive seasons. During the budgeting period, public health advocates deployed crisis- and urgency-framing messaging to target policymakers. This is significant because the Finance Act, 2021, enacted the 10-naira-per-litre tax on SSBs. A Nigerian government’s legislation enacted during the budgetary period. However, industry and its allies used messages of economic doom, unemployment, and the strain on Nigeria’s public finances to advance their position against the tax in this period. According to Kingdon [57], policy windows are opportunities in which policy entrepreneurs can present challenges with viable policy solutions to advance agenda-setting. In this period, the policy equilibrium is unsettled, reflecting the dynamics of punctuated equilibrium as espoused by Jones & Baumgartner [18].

As stated above, both public health advocates and industry supporters deployed the discursive elements of doom and urgency; they seem to navigate the policymaking waters in anticipation of policymakers’ shifting preferences [52] for policy solutions based on emergency and available information. Note that the general claims of excessive sugar consumption through SSBs have been linked to an information gap. In promoting taxes as an economic model for public health interventions, information asymmetry plays a major role. It is therefore significant to examine how campaigners and opponents use information.

The identified festive season (mainly in December, when more Nigerians are in the ‘owambe’ mood) in Nigeria coincides with the observed budgetary period, thereby extending the period of intensive media engagements to sustain the arguments of both sides. By deploying social good and morality elements, public health advocates focus on healthy consumption and self-preservation, as well as collective national health outcomes. On the other side, allies of the SSB industry and business concerns wield the double-edged sword of government intervention, stifling personal choices and thereby truncating the celebration of citizens. At the same time, the steep decline in sugary drink sales will affect distributors and retailers of these products.

Although we have consistently referenced industry allies, it is important to note that public health advocates also speak through allies. This representation demonstrated the strength of allyship and was supported by a broad range of media spokespersons, including the Health and Finance ministries, the Customs service, and others. There are also medical professionals, child rights campaigners, public health campaigners, policy experts, and nutritionists speaking in support of the tax from a position that reflects the CSOs’ position. However, the opposition was represented only by affiliates of their businesses and front groups.

‘One of the major focuses of the Sugar-Sweetened Beverages Tax Coalition is to see the government put in place a framework that will allow it to take the tax and use it appropriately for public health’ [102]. The comment by Barnabas Yahaya, a pharmacist and member of the National Sugar Sweetened Beverages Tax Coalition, showed that there is a strong, cohesive front of CSOs supporting the government’s pro-public-health tax initiatives, with other CSOs leading the charge. Leveraging the power of a coalition or movement with similar ideas in this manner aligns with Sabatier’s thinking about policy implementation. According to Sabatier, the Advocacy Coalition Framework (ACF) describes how policy actors, in various forms, advance their agreed-upon problem definitions and solutions for adoption and, therefore, implementation. It presupposes that coalitions seeking to translate their beliefs into policies would compete within a policy subsystem by employing different strategies to influence government decision-makers [79]. Sabatier and Hanks noted that with "wicked" problems – those involving substantial goal conflicts, important technical disputes, and multiple actors from several levels of government are likely to be confronted by different ideas, using similar strategies or otherwise. Advocacy Coalitions can be subsystems within Kingdon’s policy communities. However, they are usually not to be confused with ‘problem entrepreneurs’ whose trade in stock is the definition of problems without solutions, nor the ‘policy entrepreneurs’, those who wait in the corridors of policy discussions with ready-made solutions for any form of problem. The advocacy coalition proffers a solution, but from the standpoint of their agreed interests, including the definition of terms, arrived at by consensus and alignment.

In terms of themes and discourse elements employed by the coalition of public health advocates, they combined the public health messaging with governance and legacy framing. Their demand for a review of the tax and a proposal to earmark it signifies the inefficiency of the existing tax. It also raises an important issue in public trust or mistrust, as the case may be, that could be resolved through earmarking for health. The issue of earmarking is also raised tangentially by representatives of the SSB industry and its allies. Though they downplay the public health issues, they raise concerns about accountability when the tax collected on SSB is not channelled to healthcare, the primary reason government and public health advocates cite for initiating the tax.

**Fig. 4:**
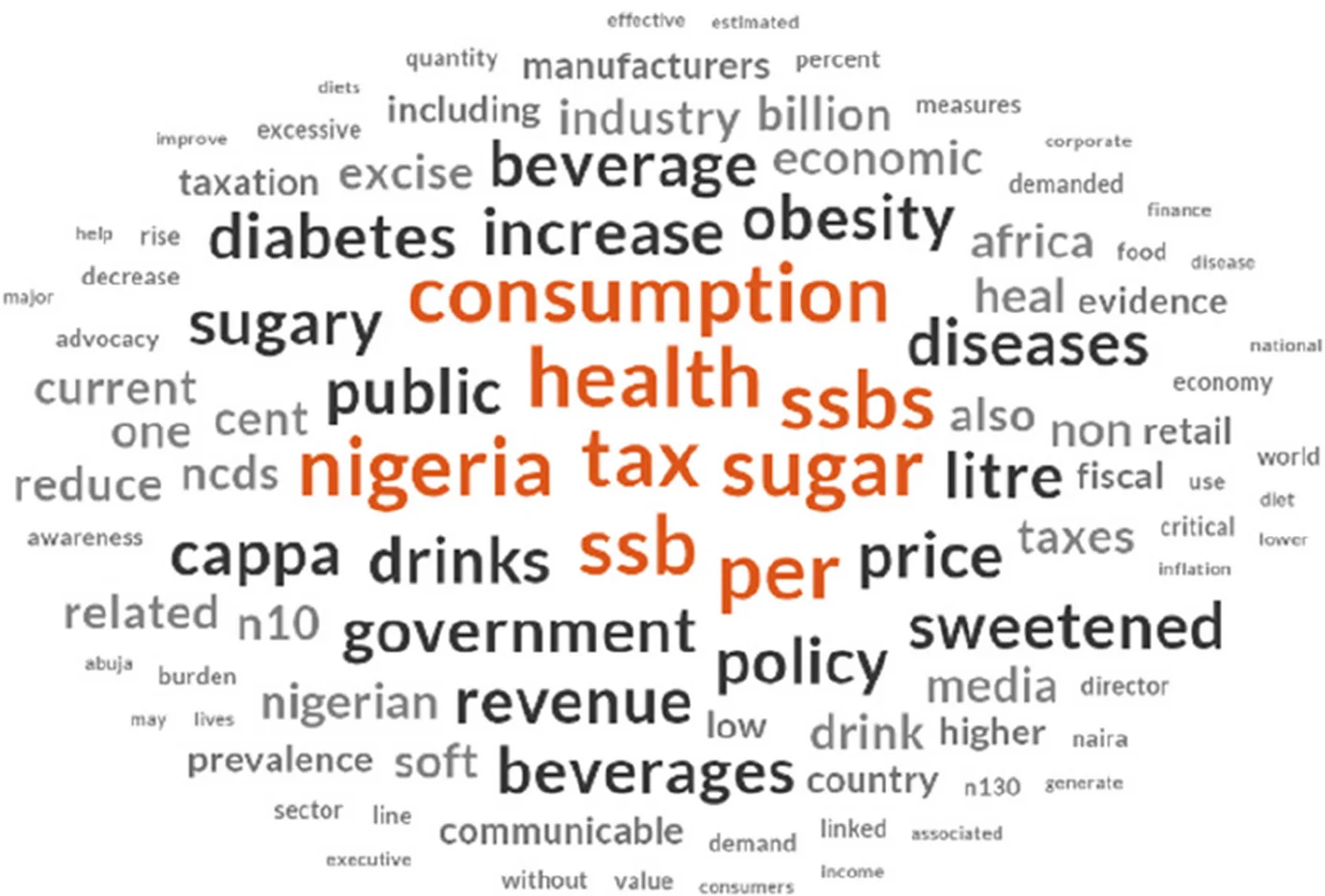
Thematic Word Cloud. *Source: NVivo Data Analysis of Media Links*

In the Nigerian case of low sugar consumption, the purveyors of the data deliberately ignore public data on excessive sugar consumption and diabetes, bringing in a sector that is not directly impacted by the SSB tax. This was also seen in industry’s preference for ‘sugar tax’ as against the ‘Sugar Sweetened Beverages tax’ widely used by public health advocates and government officials. See Fig. 4 for commonly used words along with themes of discussion. This is akin to the approach used by observed industry players in public health campaigns, who deliberately mix themes, topics, and information to mislead the public. Using the example of the tobacco industry, the Truth Project confirmed that their distortion of public information and health data is not a coincidence. This attribute has effectively been passed from big tobacco to big food [21, 22, 28].

Generally, the industry response was primarily one of confrontation and defence, as evidenced by the title: *"MAN Firebacks at NGOs Advocating for SSB Tax Hikes".* As seen in the press statements, opinion articles, and media releases by manufacturers of soft drinks and their allies, they forecasted economic downturn, loss of jobs, counterfeiting, smuggling of unregistered and illegal products, and sometimes denounced the causal effects of SSB consumption to Obesity, Diabetes, and other associated diseases while also putting the burden of responsibility on the consumer. The imagery in their media presentation suggests a weak fiscal future for Nigeria and for consumers in vulnerable groups, who they said are enjoying SSBs as a staple [3].

**Fig. 5:**
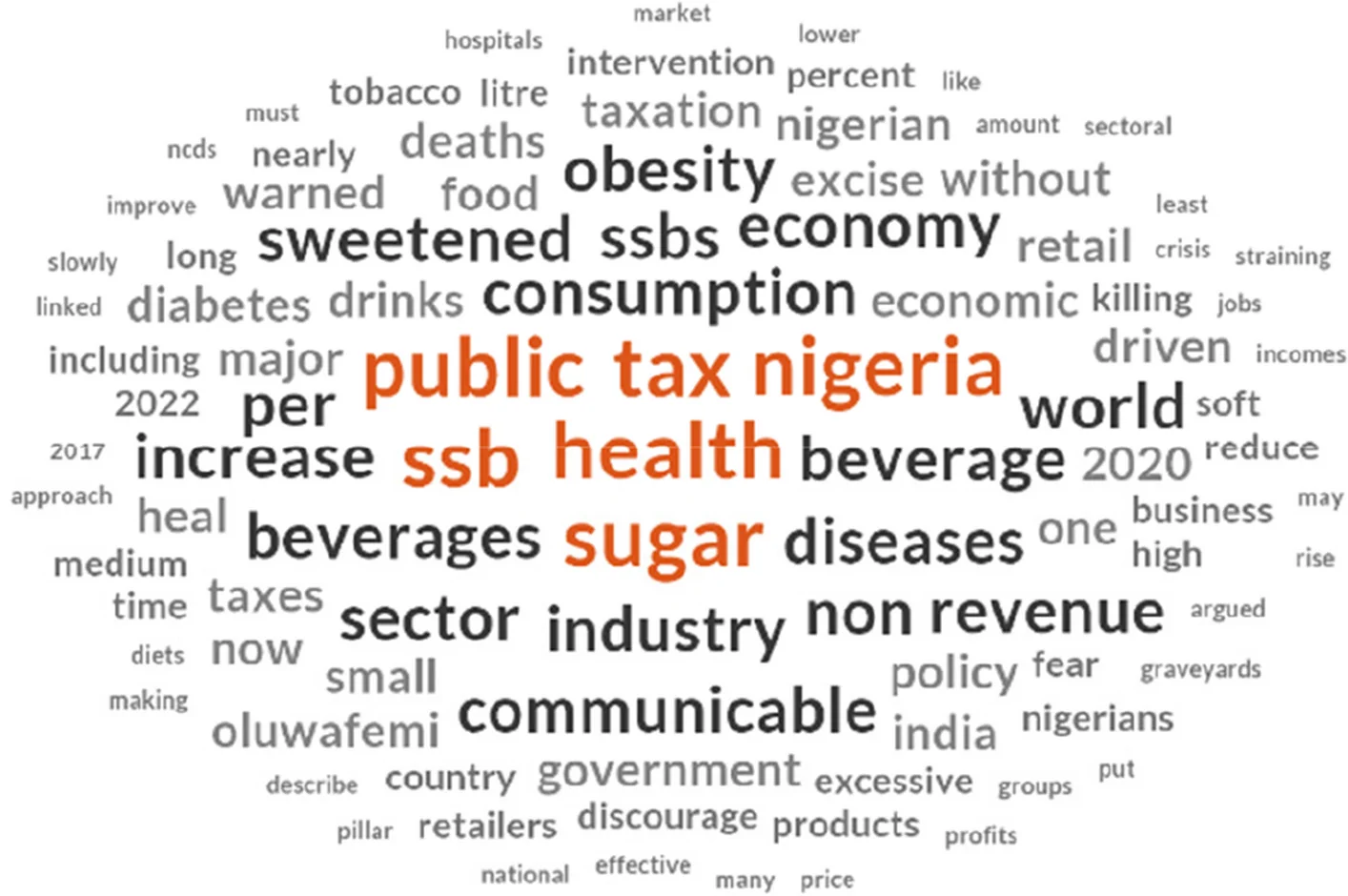
Discourse Word Cloud. *Source: NVivo Data Analysis of Media Links*

In strengthening opposition’s view against the debilitating impact of SSBs consumption, allies and front groups associated with the producers of SSBs attacked the public health coalition advancing the pro-health conversation, saying that *‘the recent call for the SSBs Tax increase is being fueled by CAPPA’*, whose argument for the tax and its increase is anchored on SSBs being an affordable and broadly available means of consuming excess sugar that is injurious to health [6]. In Fig. 5, common words deployed along discursive elements are shown as a word cloud.

To this extent, concerns about the determinants of health, both commercial and social, are widely debated and contested in public health discourse. The Commercial Determinants of Health (CHoD) refers to strategies and approaches used by the private sector to promote products and choices primarily driven by profit, often at the expense of public health [97]. These activities can influence health outcomes both positively and negatively, shaping public behaviour and policy through marketing, lobbying, and supply chain decisions [50].

While CHoD actors contribute positively, SSB producers fall within the negative scope. They often employ aggressive marketing tactics that promote unhealthy diets, particularly among vulnerable populations, thereby exacerbating health inequities and undermining national health outcomes [34].

The Social Determinants of Health (SDoH) examine the broader conditions in which people are born, grow, live, work, and age, including access to quality education, economic stability, housing, and healthcare [65]. These factors are critical to shaping health outcomes at both individual and societal levels.

In many cases, negative commercial determinants often exploit the gaps in social determinants. For example, in communities with limited access to education or healthcare, aggressive marketing of unhealthy products can disproportionately affect those least equipped to make informed health decisions, effectively trapping them in a cycle of poor health outcomes driven by commercial interests [56].

Of particular note is the claim that such narratives affected the economy, primarily because businesses sought revenue. The economic claims include the purchase of substandard products by poor households [13], job losses and the collapse of SSB production industries [20], and dwindling government revenue [93]. This type of narrative is consistent with the big food and big tobacco industries’ playbook, as seen in response to mandatory regulations and taxes on alcohol and tobacco [14]

## Conclusion

The categories of stakeholders identified in this body of work fundamentally employed different framing strategies that directly compete for public and political legitimacy [4, 47, 78]. The framing of the conversation surrounding the SSB tax observed in this body of work can be categorised into two major themes: Health and Economic benefits. On the one hand, those who support taxes are demanding better budgetary allocation to health and protection of vulnerable groups from incurring higher healthcare costs. On the other hand, the opposition denies health impacts, justifies job security, and reiterates profit over public health and the economic sustenance of the public and the country.

**Fig. 6:**
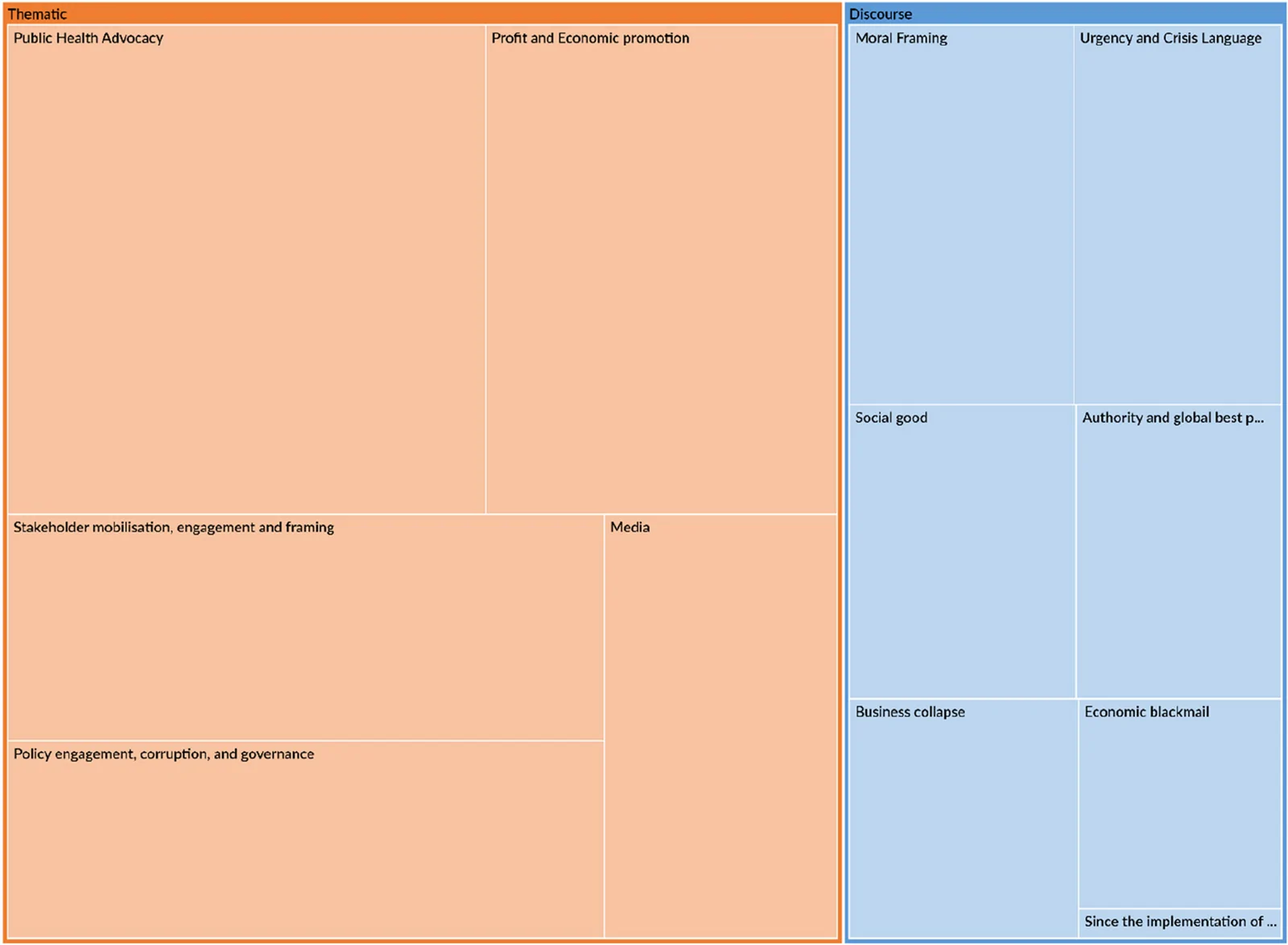
Heat Map of Coded Nodes. *Source: NVivo Data Analysis of Media Links*

The heat map, which shows a graphical distribution of themes and discursive elements, is presented in Fig. 6.

Public Health Advocates relied on Moral Framing and Urgency/Crisis Language, complemented by public health themes, positioning the tax as a life-saving measure supported by global best practices (WHO). Their goal is to shock the public with the crisis factor and pressure the government into taking action to retain and increase the SSB tax.

The SSB Industry allies use Authority to delegitimise the data put forth by public health advocates and their coalition, simultaneously deploying a counter-frame of Economic Social Good to portray the tax as a threat to national employment and economic stability.

Media Reporting is predominantly Neutral and Technical, focusing on Policy Mechanics (elasticity, tax rate effects) and neutrally quoting competing claims to global authority (WHO/International data), acting as a conduit for the debate rather than a dominant framer. There were also indications that some journalists focused on select stakeholders and rehashed their preferred narratives, but the reporting mode balanced views, reinforcing their roles as conduits rather than primary framers of the conversation.

The evidence suggests that for the tax to be more effective, there must be greater transparency in the Policy Engagement theme, particularly regarding revenue earmarking, which is currently a weakness in the discourse. This weakness was acknowledged by all stakeholders, even when it was used differently to support talking points and arguments. Transparency in the broader conversation about SSB taxes goes beyond the tax’s application; it also encompasses its objectives, stakeholder engagement, and governance. UNICEF and WHO, alongside other supranational organisations, advised that taxes on SIN products, including SSBs, should be implemented as part of a multi-pronged approach that includes public awareness.

The public awareness component further positions the media as critical to a successful SSB tax, underscoring the need for a strategic engagement plan by government and public health advocates.

## Recommendations

Based on the thematic and discourse findings, the following recommendations are offered to strengthen public health policy, advocacy, and government action. Public health advocates, CSOs, and their coalitions need to diversify the "Social Good" Frame. While the health crisis frame is effective, advocates should strengthen the economic component of the Social Good discourse. This can be achieved by publicising the win–win-win principle. This principle is credited to WHO as the organisation posited that a practical SSB tax benefits in three ways: ‘a win for public health by reducing consumption of sugary drinks, a win for government revenue that can be used for public services or health programs, and a win for health equity, primarily when taxes are structured to benefit lower-income communities and fund initiatives that support them. Such a narrative reframes social-reasonable discourse, and they must move beyond health outcomes to aggressively promote and demand the earmarking of a portion or all of the revenue generated by the tax. If this earmark is done successfully, advocates must also commit to tracking the use of earmarked revenue for public programs (e.g., school feeding, basic health services), directly countering the industry’s economic threats. Therefore, this action actively shifts the focus from *increasing taxes to demonstrating the tangible benefits of the revenue collected*.

Upon establishing the selective usage of authority framing by industry allies, health advocates must consider Proactive Data Counter-Framing. Advocates for Public Health should be prepared to immediately and publicly counter any industry attempts to use selective Authority data (e.g., low per capita sugar consumption) by contextualising it in relation to local consumption patterns and health indicators (e.g., NCD rates).

Representatives of government and other interested policymakers must work to use the media more and to own the narrative. Their engagement must go beyond headlines, but as symbolic supporters of the tax or otherwise, if the government’s position changes. This public-facing engagement could be pivotal in securing organic media support without the contestation between public health advocates and industry players. It will also provide a platform for policy influencers to strengthen the messages and framings that resonate with the public when leading the public awareness campaign.

To improve acceptance of the tax as a public health tool, the government must establish a Transparent Earmarking Mechanism. The analysis showed a gap in the Policy Engagement, Corruption, and Governance theme regarding revenue utilisation. Both industry actors and public health advocates decried the lack of public information about the revenues generated. The industry allies used this as a point to discontinue the tax and focus on other interventions that do not include taxes. However, health campaigners used the lack of public data to advance their demand for a higher tax [91] and a review of the entire tax structure. The Government must make a public, verifiable commitment to earmark revenue and release regular reports on its receipts and allocations. This will legitimise the tax in the eyes of the public and the media, build public trust, and likely strengthen the nudge towards behavioural (dietary) change. It will also deprive the industry of a key line of attack (lack of transparency) while citing other government tax-related reforms without accountability. It will improve the general acceptance of other taxes. This can be achieved by creating a dedicated, publicly accessible dashboard tracking SSB tax revenue and expenditure on health initiatives.

Furthermore, government agents and other key policymakers must reinforce the WHO Authority Frame. Policy communications should consistently link the SSB tax to the World Health Organisation’s (WHO) global best practices and to other global authorities supporting the intervention, to reinforce its image as a technically sound, evidence-based health policy rather than just a revenue-generating tool.

As for media players, they must strive to balance the Technical and Human Story angles. As seen in the analysis, the media effectively covered Policy Engagement (price elasticity, tax rates), future reporting should ensure that the Moral Framing (human cost of NCDs) is balanced with the Economic Social Good (job/sector impact), presenting a holistic view of the policy’s trade-offs. While these trade-offs have been effectively dismissed by research showing no negative impact of the SSB tax, it is still important for the media to spotlight this and ensure that the commercial determinants of health do not hold sway, pushing profit over health. As part of their role as the 4th estate of the realm, journalists and the media must go beyond quoting stakeholders to commission independent reports on the economic impact vs health cost savings; state/burden of diseases, and the true nature of sugar-sweetened beverages.

Though this research has gaps in its ability to determine how the media discourse impacted consumers’ ability to make immediate decisions, government officials, lawmakers and other stakeholders can conclude that the deployment of the health narratives can promote wider and people-driven support, while arguments focusing on profit and economic narratives, as pushed directly and indirectly by producers of SSBs and their allies can work against the objectives of the tax.

This work contributes to the body of knowledge on media analysis of the SSB tax, providing a robust foundation for more detailed analysis of content and networks.

## Data Availability

The data that support the findings of this report are available from the corresponding author, [A S], upon request.

## Abbreviations

ACF: Advocacy Coalition Framework
APH: Advocates and Public Health
AIT: African Independent Television
CDC: Centres for Disease Control and Prevention
CSEA: Centre for the Study of Economics of Africa
CIFIAN: Chartered Institute of Financial and Investment Analysts, Nigeria
CSOs: Civil Society Organisations
CHoD: Commercial Determinants of Health
CAPPA: Corporate Accountability and Public Participation Africa
DA: Discourse Analysis
FCE: Federal Government College of Education
FUNAAB: Federal University of Agriculture, Abeokuta
GDP: Gross Domestic Product
GP: Government and Policy
IA: Industry and Allies
MAN: Manufacturers Association of Nigeria
MSR: Media Source Reporting
MAPOLY: Moshood Abiola Polytechnic
MMTQDA: Multi-Method Qualitative Text and Discourse Analysis
NSSBTC: National Sugar Sweetened Beverages Tax Coalition
NUFBTE: National Union of Food, Beverage and Tobacco Employees
NCD: Non-communicable Diseases
SDoH: Social Determinants of Health
SSBs: Sugar-Sweetened Beverages
TA: Thematic Analysis
TGNC: Transgender and Gender Non-conforming
UNICEF: United Nations Children’s Fund
WHO: World Health Organisation
LMICs: Low- and Middle-Income Countries
